# Parallel Bayesian Optimization of Thermophysical Properties of Low Thermal Conductivity Materials Using the Transient Plane Source Method in the Body-Fitted Coordinate

**DOI:** 10.3390/e26121117

**Published:** 2024-12-20

**Authors:** Huijuan Su, Jianye Kang, Yan Li, Mingxin Lyu, Yanhua Lai, Zhen Dong

**Affiliations:** 1 School of Nuclear Science, Energy and Power Engineering, Shandong University, Jinan 250061, China; 2 New Energy and Environmental Protection Technology Innovation Platform, Suzhou Research Institute of Shandong University, Suzhou 215123, China

**Keywords:** thermophysical properties, transient plane source method, body-fitted coordinate system, multi-objective hybrid strategy, parallel Bayesian optimization

## Abstract

The transient plane source (TPS) method heat transfer model was established. A body-fitted coordinate system is proposed to transform the unstructured grid structure to improve the speed of solving the heat transfer direct problem of the winding probe. A parallel Bayesian optimization algorithm based on a multi-objective hybrid strategy (MHS) is proposed based on an inverse problem. The efficiency of the thermophysical properties inversion was improved. The results show that the meshing method of 30° is the best. The transformation of body-fitted mesh is related to the orthogonality and density of the mesh. Compared with parameter inversion the computational fluid dynamics (CFD) software, the absolute values of the relative deviations of different materials are less than 0.03%. The calculation speeds of the body-fitted grid program are more than 36% and 91% higher than those of the CFD and self-developed unstructured mesh programs, respectively. The application of body-fitted coordinate system effectively improves the calculation speed of the TPS method. The MHS is more competitive than other algorithms in parallel mode, both in terms of accuracy and speed. The accuracy of the inversion is less affected by the number of initial samples, time range, and parallel points. The number of parallel points increased from 2 to 6, reducing the computation time by 66.6%. Adding parallel points effectively accelerates the convergence of algorithms.

## 1. Introduction

With the rapid development of thermal insulation materials, higher requirements are put forward for the measurement of thermal properties of thermal insulation materials [1]. The heat transfer properties of materials are closely related to their thermophysical properties, such as thermal conductivity, specific heat capacity, and thermal diffusivity. The transient plane source method is a commonly used experimental measurement of thermal conductivity and thermal diffusivity, which is widely adopted to measure the thermal conductivity of bulk, thin slab, and film samples [2,3]. The key to the transient plane source (TPS) method is the probe, which has both temperature measurement and heat source functions [4]. The conventional TPS probe applies an etching process to obtain a double spiral structure. During the actual measurement, the probe is placed between two pieces of samples and heated, and the temperature increase is used to reflect the samples’ thermophysical properties [5]. The TPS method simplifies the actual model to obtain many ideal models [6,7,8], but the difference between the actual structure and the ideal model leads to measurement error, which is affected by the shape of the probe and the thermophysical properties of the sample. In order to approach the actual model, many scholars studied the various factors simplified in the ideal model to improve the solution accuracy [9,10,11,12].

In practical engineering applications, it is often encountered that the thermophysical parameters cannot be obtained by direct contact with the material under test, and the thermophysical parameters of the materials can only be estimated by temperature values and other known conditions, which is called the inverse problem of heat conduction [13,14]. It is difficult for the traditional analytical model of the TPS method to deal with the complex heat transfer problems caused by actual physical phenomena [15]. Numerical methods are more suitable to solve such complicated heat transfer problems, but a single numerical model cannot directly estimate the parameters. To find the optimal parameter solution that corresponds to the minimum difference between the analytical response of the direct problem and the known data, an optimization algorithm is required, based on the inverse problem theory in heat transfer [16].

Parallel Bayesian optimization methods are proposed by many scholars to shorten time. The aim of adding parallel points is to enrich the diversity of parallel points and maximize the benefits. Ginsbourger [17] improved q-EI by maximizing multi-point expectations to achieve parallel evaluation, together with Chevalie et al. [18], who further introduced heuristic methods to jointly evaluate q-EI’s maximized point set. Shahriari [19] and Ghahramani [20] generated parallel points through the method of parallel predictive entropy search. Nguyen et al. [21] proposed Budgeted Batch Bayesian Optimization, which used an infinite Gaussian mixture model to fit the acquisition function and selected the average value of each Gaussian component as a parallel point. Ji et al. [22] proposed a Bayesian optimization algorithm with an adaptive initial population, which identified the thermal conductivity of solid samples.

Compared with the traditional algorithm, this paper improves the solving speed from two aspects: direct problem solving and inverse problem optimization. Considering that in the process of numerical solution, Mohebbi and Sellier [23,24] adopted BFG generation to deal with the unknown boundaries and mapped the three-dimensional irregular physical domain to a regular (cuboid) computing domain, which greatly reduced the computational time. This paper first considers the combination of a body-fitted coordinate generation technology and the TPS method to realize the grid structure of the physical model. Different from the previous schemes, an inverse problem introduces multi-objective optimization and probability to address the problem of adding parallel points. The strategy of adding points is integrated with multi-objective integration.

The main works addressed in this article are as follows: (1) A direct problem model of TPS heat conduction based on body-fitted mesh is constructed. (2) An inverse problem optimization model of the TPS heat conduction model is established, using a Pareto frontier parallel Bayesian optimization algorithm. (3) The division angle of body-fitted mesh is analyzed, and the mesh independence, time step independence, correctness verification, efficiency, and sensitivity of the direct problem are studied. The inverse problem benchmark function is optimally selected. The effects of different time ranges, the number of initial samples, and parallel points on the results of inversion are investigated.

## 2. Model

### 2.1. Heat Transfer Physical Model

**Figure 1:**
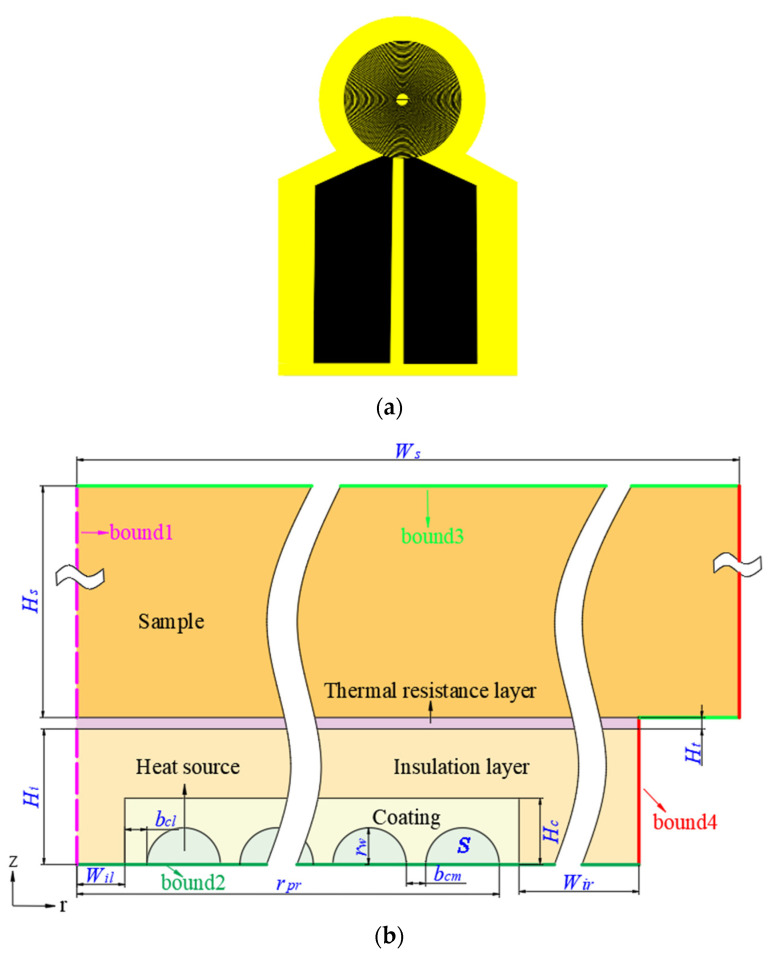
The physical and geometric model. (**a**) The probe geometric model; (**b**) the TPS probe and sample physical model.

The probe geometric model is shown in Figure 1a. The new probe winds in the same plane using the 0.04 mm wire-enameled wire, and the epoxy resin on the outside is melted by high-temperature heating and solidified after cooling to achieve a self-adhesive setting. The probe and sample physical model are shown in Figure 1b, simplifying the three-dimensional model into a two-dimensional axis-symmetric model, including the heat source, coating, insulation layer, thermal resistance layer, and sample domain. The heat source material is copper wire, to which constant power is applied, and heat is dissipated to the rest. In the actual heat transfer process, there is thermal contact resistance between the parts of the probe, which is attached to the thermal contact resistance between the insulation layer and the sample during simulation, and the thermal contact resistance applies the equivalent thin-wall model [25]. Low thermal conductivity materials are selected in this paper, including sample glass wool (GW), polystyrene foam (PSF), and extruded polystyrene (XPS). The detailed geometric parameters and the thermophysical properties of the probe and sample are listed in Table 1.

The thermal resistance layer is set in the physical model, and the thermal contact resistance is changed by changing the thickness of the thermal resistance layer. In order to emphasize the effect of the thermal contact resistance, the thickness is selected to be 0.038 mm. The relationship between the thickness of the thermal resistance layer and the thermal contact resistance is as follows [26]:

(1) $$hc=\delta_{t}/\lambda_{t}$$

where hc is the thermal contact resistance, $\delta_{t}$ is the thickness of the thermal resistance layer, and $\lambda_{t}$ is the thermal conductivity of the thermal resistance layer.

### 2.2. Heat Transfer Mathematical Model

The model is reduced to a two-dimensional axisymmetric heat conduction differential equation. The model has cylindrical symmetry. Using a cylindrical coordinate system can save calculation and speed up the solution. The heating time is chosen to ensure that the heat flow does not reach the outer boundaries of the sample [12], and the outer boundaries of the sample are set as adiabatic edges. The heat flow distance is calculated according to the following formula [12]:

(2) $$\Delta h=2\sqrt{\kappa_{s}t_{\mathrm{tol}}}$$

where Δh is the heat flow distance, $\kappa_{s}=\lambda_{s}/(c_{ps}\rho_{s})$ is the diffusivity of the sample, and $t_{\mathrm{tol}}$ is the total heating time.

During the heating process, the final probe temperature rise should be less than 5 K to satisfy the assumption of constant thermophysical properties of each material [27]. The thickness of the probe is in the micron scale, and the thermal conductivity of the air is small, which plays an adiabatic role on the outer boundary of the probe. The outer boundary of the insulation layer is the adiabatic edge. The specific mathematical model is shown in Table 2.

### 2.3. Transformation of Body-Fitted Mesh Model

#### 2.3.1. Mesh Partitioning

Meshing is mainly divided into structured and unstructured meshes. The calculation cost of numerical model division of structured meshes is lower than that of unstructured meshes [29]. While unstructured meshes are more adaptable to complex geometric regions but slower in computation. BFG combines the advantages of both, is computationally faster than unstructured meshes, and can be adapted to irregular boundaries. In this paper, the solution region is simplified by constructing the body-fitted coordinate system fitted to the complex boundaries, and the coordinates of the unstructured mesh in the physical plane are transformed into the coordinates of the structured mesh in the computational plane.

**Figure 2:**
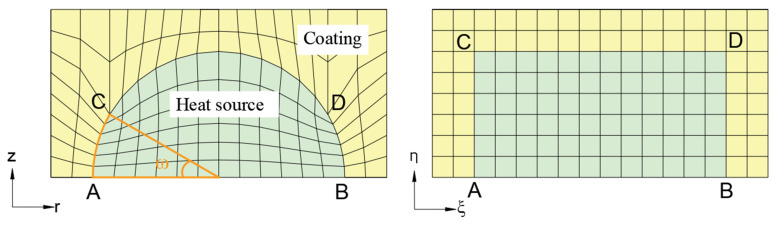
The coordinate transformation schematic diagram of BFG.

BFG is applied to divide irregular domains with circular boundaries such as heat source and coating; regular domains, such as insulation layer and sample, are delineated into structured meshes. Four points on the circular boundary of the heat source are selected as the dividing basis, denoted as A, B, C and D, respectively. Two points, C and D, are selected for grid division according to different angles. Figure 2 is a schematic diagram of the transformation of the BFG coordinates in a 30° division mode, converting them from the physical plane to the computational plane.

Calculate the differential equation on the plane as follows [24]:

(3) $$\begin{array}{c} \alpha_{0}r_{\xi \xi }-2\beta_{0}r_{\eta \xi }+\gamma r_{\eta \eta }=-J^{2}(Pr_{\zeta }+Qr_{\eta })\\ \alpha_{0}z_{\xi \xi }-2\beta_{0}z_{\eta \xi }+\gamma z_{\eta \eta }=-J^{2}(Pz_{\zeta }+Qz_{\eta }) \end{array}$$

Parameters in the formula are defined as [29]:

(4) $$\begin{array}{c} \alpha_{0}=r_{\eta }^{2}+r_{\eta }^{2}\\ \beta_{0}=r_{\xi }r_{\eta }+z_{\xi }z_{\eta }\\ \gamma =r_{\xi }^{2}+z_{\xi }^{2}\\ J=r_{\xi }z_{\eta }-r_{\eta }z_{\xi } \end{array}$$

where ξ and η are the horizontal and vertical coordinates in the computational plane, respectively. $\alpha_{0}$ and γ are the metric coefficients in the η and ξ directions, respectively. $\beta_{0}$ reflects the orthogonality of physical plane grid, reflecting the intersection angle of two grid lines, and J is the Jacobi factor. P,Q are control functions used to adjust the grid distribution and the orthogonality of the grid in the region, where the region shape is relatively simple, so that P=Q=0.

#### 2.3.2. Transformation and Discretization

The governing equation corresponding to the domain of the BFG should be transformed. Converting the governing equations from the physical plane to the computational plane [30], the transformed mathematical model is:

(5) $$\rho c_{p}\frac{\partial T}{\partial t}=\frac{1}{rJ}\frac{\partial }{\partial \xi }\left[\frac{r\lambda }{J}(\alpha_{0}\frac{\partial T}{\partial \xi }-\beta_{0}\frac{\partial T}{\partial \eta })\right]+\frac{1}{J}\frac{\partial }{\partial \eta }\left[\frac{\lambda }{J}(\gamma \frac{\partial T}{\partial \eta }-\beta_{0}\frac{\partial T}{\partial \xi })\right]+R(\xi ,\eta )$$

where R is the heat source term in the computational plane.

The transient term is discretized as:

(6) $$\int_{s}^{n}\int_{w}^{e}\int_{t}^{t+\Delta t}r\rho c_{p}\frac{\partial T}{\partial t}dtd\xi d\eta =r_{p}\rho c_{p}(T_{P}-T_{p}^{0})\Delta \xi \Delta \eta$$

where subscript p represents the center of the solution unit. The superscript 0 represents the last time step. n, s, e, and w represent the upper, lower, right, and left edge centers of the solution grid, respectively.

The diffusion term is discretized [28,31]:

(7) $$\begin{array}{l} \int_{s}^{n}\int_{w}^{e}\int_{t}^{t+\Delta t}r\frac{1}{J}\frac{\partial }{\partial \xi }\left[\frac{\lambda }{J}(\alpha_{0}T_{\xi }-\beta_{0}T_{\eta })\right]dtd\xi d\eta +\int_{s}^{n}\int_{w}^{e}\int_{t}^{t+\Delta t}r\frac{1}{J}\frac{\partial }{\partial \eta }\left[\frac{\lambda }{J}(\gamma T_{\eta }-\beta_{0}T_{\xi })\right]dtd\xi d\eta \\ =\frac{1}{J}\left[D_{e}(T_{E}-T_{P})-D_{w}(T_{P}-T_{W})\right]\Delta t-\frac{1}{J}{\left[r\frac{\beta_{0}\Delta \eta }{J}\lambda T_{\eta }\right]}_{w}^{e}\Delta t\\ +\frac{1}{J}\left[D_{n}(T_{N}-T_{P})-D_{s}(T_{P}-T_{S})\right]\Delta t-r_{p}\frac{1}{J}{\left[\frac{\beta_{0}\Delta \xi }{J}\lambda T_{\xi }\right]}_{s}^{n}\Delta t \end{array}$$

where $D_{e,w,n,s}={\left(r\frac{\alpha_{0}}{J}\lambda \frac{\Delta \eta }{\Delta \xi }\right)}_{e,w,n,s}$. The subscripts N, S, E, and W represent the center of the upper, lower, right, and left units of the solved grid, respectively.

The source term is discretized as:

(8) $$\int_{s}^{n}\int_{w}^{e}\int_{t}^{t+\Delta t}rR(\xi ,\eta )\frac{\partial T}{\partial t}dtd\xi d\eta =r_{p}R\Delta t\Delta \xi \Delta \eta$$

The lower boundaries of the heat source and the coating are symmetric boundaries. After dividing into the BFG, the boundary condition conversion is required to convert the symmetrical boundary from the physical plane to the computational plane. The conversion to the computational plane is expressed as follows:

(9) $$\gamma T_{\eta }-\beta_{0}T_{\xi }=0$$

Equation (8) is discretized in a finite difference scheme as an additional source term [31].

## 3. Inverse Heat Conduction Problem

### 3.1. Optimization Model

The principle of inversion is based on the optimization algorithm to find the optimal parameter solution corresponding to the minimum difference between the analysis response of the direct problem and the known data. Inversion uses an optimization algorithm to find the optimal parameter solution that corresponds to the minimum difference between the analytical response of the direct problem and the known data. An objective function needs to be constructed to reflect this difference. The direct problem algorithm is used to obtain the average temperature rise in the probe heat source, and the MHS is applied to identify the thermal conductivity, specific heat capacity, and thermal contact resistance at the same time. An objective function is constructed to transform the parameter identification process into an optimization problem, as shown in Equation (10). The initial parameter range settings are shown in Table 3.

(10) $$f(\lambda ,c_{p},hc)=\frac{1}{m}{\sum_{i=0}^{m}\left[\Delta \bar{T}(t_{i})-\Delta T_{\lambda ,c_{p},hc}(t_{i})\right]}^{2}$$

where m is the number of time steps and $\Delta \bar{T}$ is the actual average temperature rise in the probe heat source, which comes from the program calculation. $\Delta T_{\lambda ,c_{p},hc}$ is the predicted average temperature rise in the probe heat source after substituting the thermal conductivity, specific heat capacity, and thermal contact resistance in the direct problem algorithm.

According to the objective function, when $f(\lambda ,c_{p},hc)$ tends to 0, the actual mean temperature rise data of the probe is infinitely close to the predicted data, indicating that the predicted parameter values are infinitely close to the real values of the sample material.

### 3.2. Constraint Condition

The direct problem heat conduction model is taken as the constraint condition, and a biased conservative convergence condition is applied to obtain highly accurate optimization results, and the convergence condition is given in the following equation:

(11) $$\left|f_{i}(x)-f(x^{*})\right|<1\times 10^{-6}$$

where $f_{i}(x)$ is the objective function value and $f(x^{*})$ is the optimal value of the objective function in the dataset.

### 3.3. Optimization Algorithm

An MHS is proposed, and its pseudocode is listed in Algorithm 1. The algorithm consists of α and β strategies. In the α strategy, LCB [32] and EI [33] are taken as two optimization objectives to construct multi-objective optimization:

(12) minimize LCB(x),−EI(x)

LCB and EI criteria cause slow convergence of the algorithm in the later stages. A separate, more convergent β strategy is set up for this problem. In the β strategy, the prediction average, μ(x), and prediction variance, σ(x), of the probabilistic surrogate model based on Gaussian process (GP) [34] are taken as two optimization objectives. In order to speed up the convergence, the algorithm is expected to show a preference towards μ(x) while avoiding the local optimal. The probability factor, ω, is introduced and set to 0.1. The points biased towards σ(x) are uniformly selected from the Pareto front with the probability of ω. This is similar to the greedy strategy [35]. The difference is that the parallel points of the β strategy are not randomly selected but uniformly selected.

**Figure 3:**
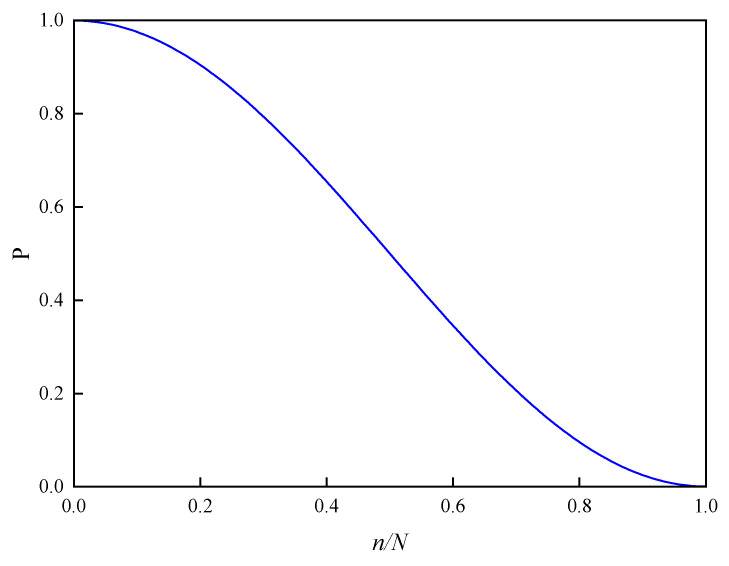
Probability selection function.

α and β strategies are combined by a probability selection function, P. Rand is a random number of 0–1. The probability function, P, shown in Figure 3, is described as:

(13) P=1−0.5(1−cos(nπ/N))

where n represents the current number of iterations, N is the maximum number of iterations set, and the function P describes the probability that the α strategy is selected.

As shown in Figure 3, the algorithm has a higher probability of choosing the exploratory α strategy in the early stage and tends to choose the more convergent β strategy in the late stage. The MHS improves efficiency and robustness while adding points, and it takes into account convergence and exploration.
**Algorithm 1** The pseudocode of MHSInitial setting:Number of initial sample M, Maximum iterations N, Decision space χ, number of parallel points PSteps:
1:Initialization2:Sample M points randomly by Latin hypercube sampling [36] in decision space χ,3:Evaluate objective function for M sample points4:Build sample database D5:Construct initial probabilistic surrogate model6:for n=1, …, N7:Construct α strategy and β strategy8:if rand < P9:Choose α strategy10:else 11:Choose β strategy12:end13:Find Pareto frontiers of α or β strategy by NSGA-II algorithm [37]14:Sample P$\;\mathrm{points}\;x_{1}\ldots x_{P}$ in the Pareto optimal solution15:Evaluate objective function for all sampling points16:Update sample database D17:Update probabilistic surrogate model18:end for19:$\mathrm{Return}\;\mathrm{optimal}\;\mathrm{sampling}\;\mathrm{points}\;x^{*}$ recorded during iteration

## 4. Results and Discussion

### 4.1. Direct Problem Analysis

#### 4.1.1. Body-Fitted Mesh Analysis

**Figure 4:**
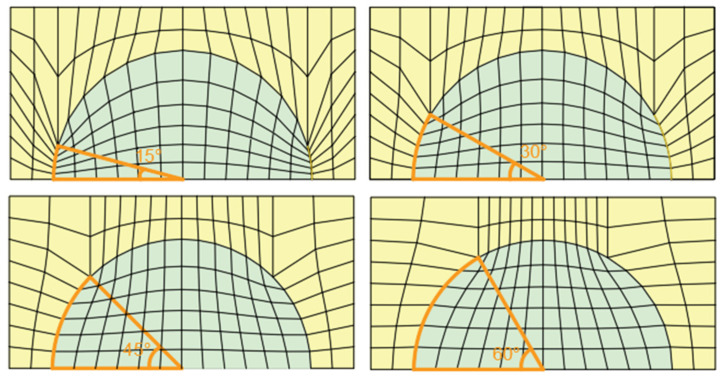
Different angles of grid division.

During the body-fitted mesh conversion process, it can be converted in different angle divisions, and angles, ω, could be selected as 15°, 30°, 45°, and 60°. The mesh division from different angles is shown in Figure 4.

**Figure 5:**
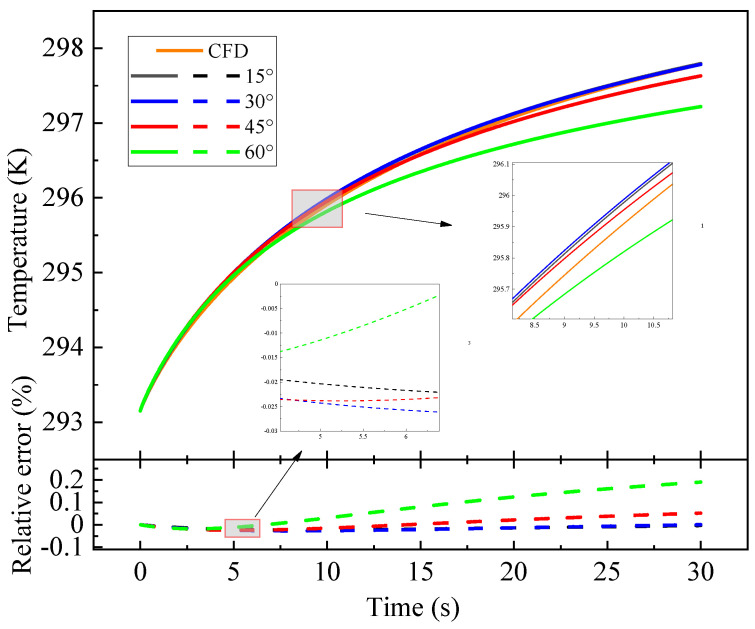
Model temperature rise at different division angles.

After regularization of relatively complex geometric regions with BFG, the average temperature rise in heat source was calculated. The influence of BFG on the temperature of heat source and coating region under different angle division forms was explored, and the calculated value was compared with that of the computational fluid dynamics (CFD). As shown in Figure 5.

When the grid is divided into 45° and 60°, the error is larger than that calculated by CFD. The reason is related to the orthogonality and mesh density of the mesh division. At 45° and 60°, the mesh density at the coating boundary is very poor, and the meshing is concentrated in the middle of the boundary. The results are close when the grid is divided at 15° and 30°. Considering the calculation cost and taking the heat source temperature appreciation at the last moment, it is found that the calculated temperature is more suitable for the CFD calculation value at 30°.

**Figure 6:**
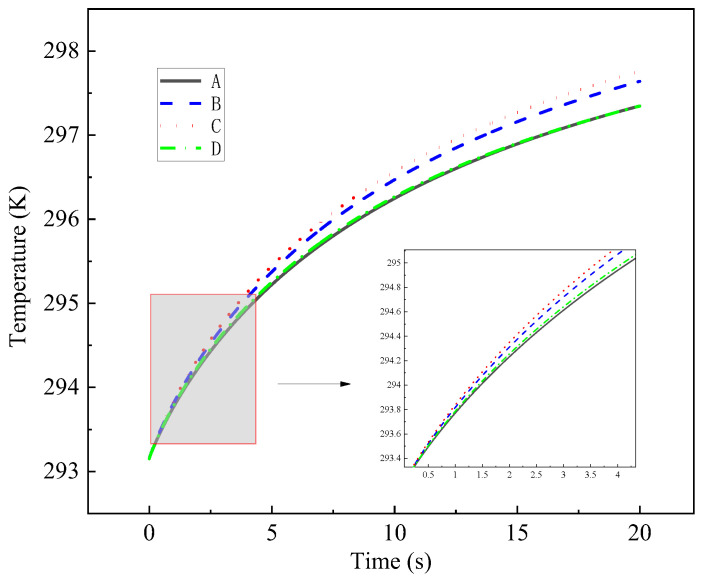
Effect of different mesh number on probe temperature rise.

The number of transverse meshing of heat sources is selected as 4, 6, 8, and 10, respectively, and other parts are consistent with the adjacent mesh size ratio. The influence of grid orthogonality and mesh density on the temperature rise in the probe during the grid transformation process was deeply analyzed. The four different grid numbers 42,844, 52,972, 63,424, and 102,507 are defined as A, B, C, and D, respectively, as shown in Figure 6. After calculation, it is found that the grid orthogonality, $\beta_{x}$, is D > A > B > C in order from large to small, and the average γ density on the boundary is B > D > C > A in order from large to small. It is found that the worse the orthogonality of the grid, the greater the temperature change, and the orthogonality of the grid plays a dominant role in the temperature change. When the number of grids is B, the mesh density on the boundary is the best.

#### 4.1.2. Grid Independence and Time Step Independence Verification

**Figure 7:**
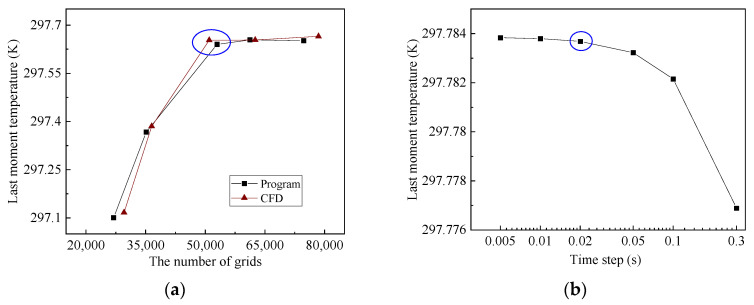
Irrelevance validation analysis. (**a**) CFD software and self-programmed grid independence validation; (**b**) time step independence verification.

The accuracy of the computational results of transient studies is related to the number of model grids and the time step. The material, XPS, was selected for grid independence validation. On the basis of the grid division angle and number of heat sources in Section 4.1, the independence verification of the self-programmed program is carried out, and the total number of grids is mainly changed by changing the size ratio of adjacent grids. The number of model grids and the corresponding temperature at the last moment of the CFD software (COMSOL Multiphysics 6.1) and self-programmed model are shown in Figure 7a. When the number of CFD software and self-programmed grids are 51,000 and 52,972 or above, respectively, the computational results are less variable. The remaining materials after verification are also selected to correspond to the number of grids.

The material GW was selected for time step independence verification to analyze the results of different time steps from 0.026 to 1 s, as shown in Figure 7b. As the step size decreases, the temperature gradually increases at the last moment, and the calculated results change less at 0.02 s and smaller steps, and the calculated time step is chosen to be 0.02 s. The time step of the remaining materials are listed in Table 4.

#### 4.1.3. Validation of the Direct Problem Algorithm

**Figure 8:**
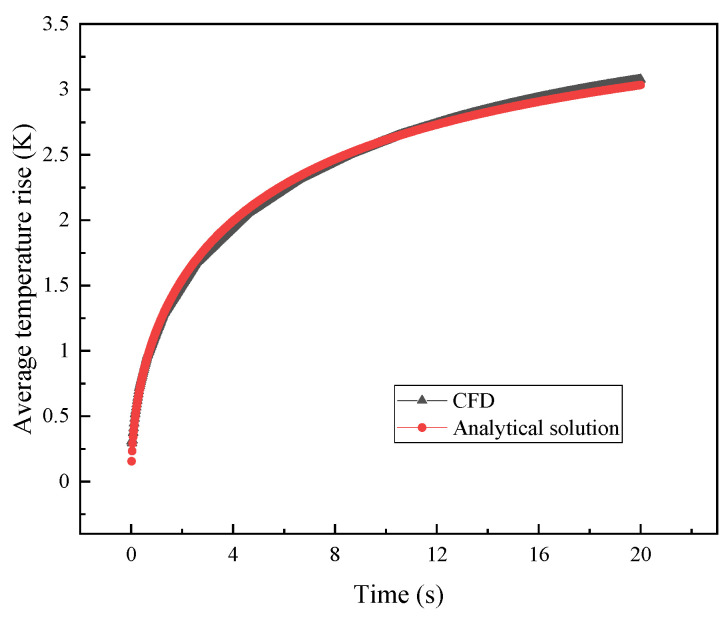
Validation of the analytical solution results and CFD software.

The finite element method used CFD software. The model uses the physical field of “heat transfer in solids”, which is discretized by quadratic Lagrange. In order to verify the reliability of CFD software results, this paper compares the analytical solution results [38] with CFD software results. As shown in Figure 8. Under the conditions of no thickness, no volume, no specific heat, and no thermal contact resistance, the average temperature rise in the probe under both methods is calculated, the coefficient of determination R^2^ is 0.9977, and the error between them is small. It indicates that the calculation results of the CFD software are consistent with the analytical solution, and verifies the correctness and reliability of the results of CFD software.

**Figure 9:**
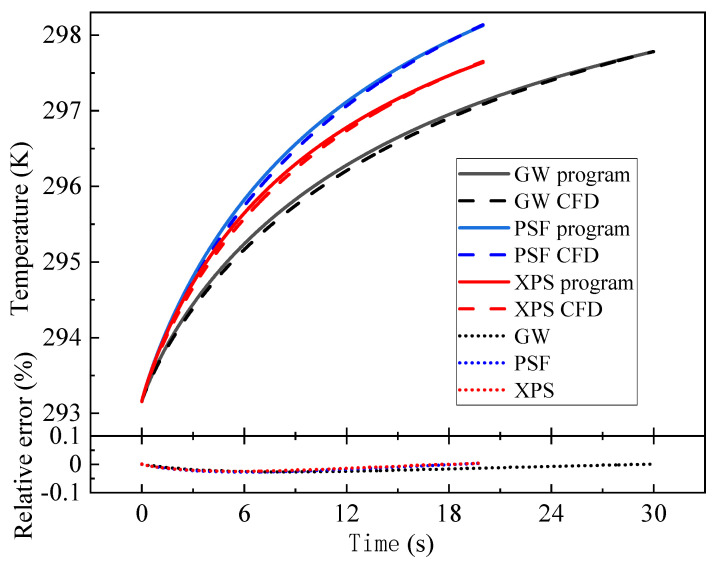
Comparison of the results for the three materials.

The direct problem algorithm was used to calculate the average temperature rise in the heat source corresponding to the three sample materials, and the results were compared with those of the CFD software to verify the correctness of the program. Both the program and CFD software were run on the same computer, the number of meshes used by the CFD software was 51,000, and the initial and boundary conditions were the same. The comparisons of the results for the three materials are shown in Figure 9. The results of the direct problem algorithm using the BFG program fit well with CFD, and the absolute values of the relative errors of both are less than 0.03% in the calculation time. It indicates that the accuracy of the program is close to that of CFD software, which can be applied to engineering calculations.

#### 4.1.4. Efficiency Analysis

**Figure 10:**
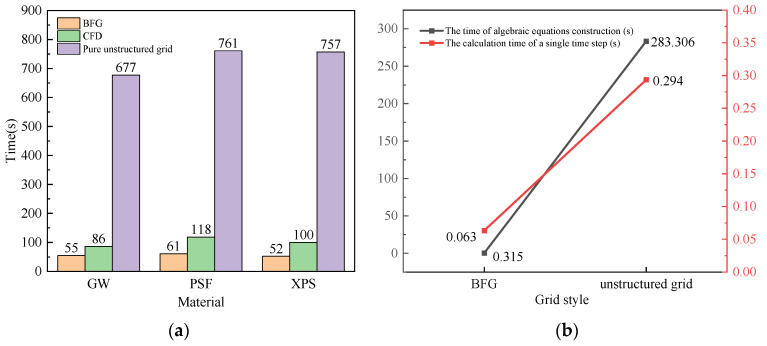
Calculation time. (**a**) Comparison of the calculated total time; (**b**) comparison of the internal calculation time of the GW materials.

Figure 10a shows the calculation time of the CFD software, the pure unstructured mesh program, and the BFG strategy. The CFD software uses the finite element method and the adaptive time stepping integral method. The total number of degrees of freedom is 159,445, and the relative tolerance and absolute tolerance are 1 × 10^−6^ and 1 × 10^−7^, respectively. The pure unstructured mesh program and the BFG strategy are both written using MATLAB R2020a software, using the finite volume method and fixed time stepping integration method. The unstructured mesh program takes longer and is computationally inefficient. For the same accuracy, the solution speed of body-fitted mesh is more than 91% and 36% higher than that of unstructured mesh and CFD software, respectively.

The GW material was selected to deeply analyze the comparison of the time processing inside the BFG and unstructured grids, as shown in Figure 10b. In the numerical solution process, after the model is meshed, the geometric and topological information from the grids is processed. Unstructured grids cannot index individual elements directly and need to traverse all cells, and the diffusion terms of the differential equations are corrected for non-orthogonality, resulting in a relatively long time for the construction of the system of algebraic equations. The structured grid coefficient matrices are based on diagonal symmetry during the computation of the individual time steps, while the unstructured grids are time consuming to solve because of the existence of non-orthogonal terms due to their non-orthogonality.

### 4.2. Sensitivity Analysis Discussion

The maximum value of the time range is obtained by sensitivity analysis. The sensitivity analysis is performed using numerical methods, and the sensitivity is calculated as [39]:

(14) $$\beta_{l}=l\frac{\partial T(\tau )}{\partial l}=l\frac{\Delta T(\tau )}{\Delta l}$$

where l represents any parameter in thermal conductivity, specific heat capacity, and thermal contact resistance, and Δl is the variation in the parameter. To consider the effects of time, the radius of probe and materials’ thermal diffusivity, and the horizontal coordinate is set to t/θ, here θ is the characteristic time defined by $\theta =r_{\mathrm{pr}}^{2}/\kappa$, and κ is the thermal diffusivity.

**Figure 11:**
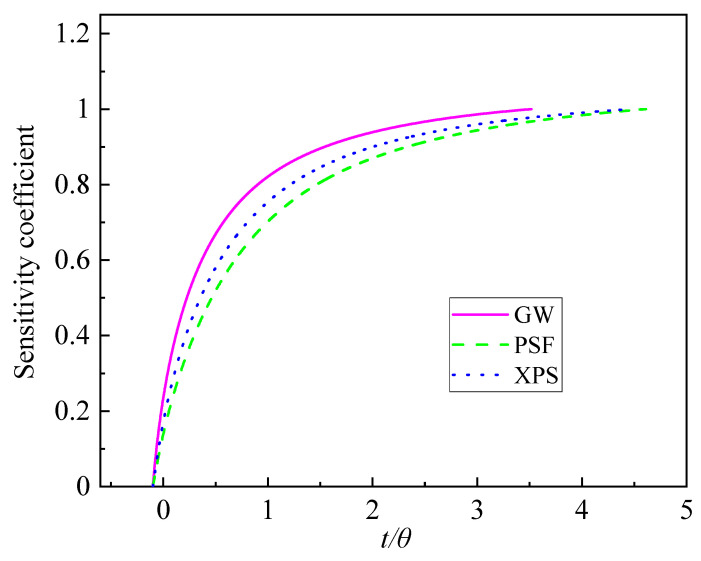
The thermal contact resistance sensitivity coefficient of the different materials.

The sensitivity of the thermal contact resistance is calculated and normalized. The closer the sensitivity coefficient to 1, the greater the influence of parameters on temperature; the closer the sensitivity coefficient is to 0, the smoother the influence of parameters on temperature. The results of the thermal contact resistance sensitivity analysis for the four materials are given in Figure 11.

From Figure 11, it can be seen that the rate of change in the sensitivity of various materials is XPS, PSF, and GW in descending order at t/θ=1. The rate of change in the sensitivity of the thermal contact resistance of other materials gradually decreases and does not go to 0, and the greater the rate of change indicates that the greater the magnitude of change in the degree of influence of thermal contact resistance on the temperature rise, the influence of thermal contact resistance should be considered in MHS, and the maximum value of the time interval selects the large part of the sensitivity change rate of thermal contact resistance, which helps to find the superior value. Table 5 lists the time intervals for different materials when using the MHS.

### 4.3. Inverse Problem Analysis

#### 4.3.1. Benchmark Function Test

The proposed MHS is tested on six benchmark functions, including Ackley, Griewank, Rastrigin, Rosenbrock, Sphere, and Step) [40,41]. Three popular multi-objective parallel Bayesian optimization methods are compared, including EI + LCB [27], greedy ϵ-PF [35], and MACE [42], which integrates multi-objective acquisition. For the MHS, EI + LCB, ϵ-PF, and MACE, a square exponent kernel is used and the hyperparameters of the GP model are fitted with maximum likelihood estimation. The dimensions and search scope of benchmark functions are shown in Table 6.

A parallel test of the algorithms is carried out. For Sphere and Step functions, the initial Latin hypercube sampling number, M, is set to 50 and the maximum number of iterations, N, is set to 100. For the other functions, M is set to 20 and N is set to 45. P is set to 4. The total number of function evaluations is M+N×P. The test is repeated ten times to average random fluctuations. The MHS is run in sequential mode and is compared with the MHS with P=4.

**Figure 12:**
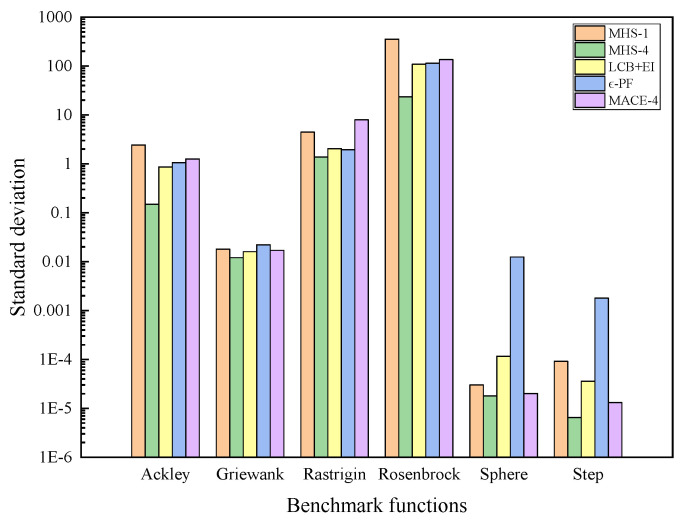
Standard deviation of the MHS, EI + LCB, ϵ-PF, and MACE for benchmark function optimization.

**Figure 13:**
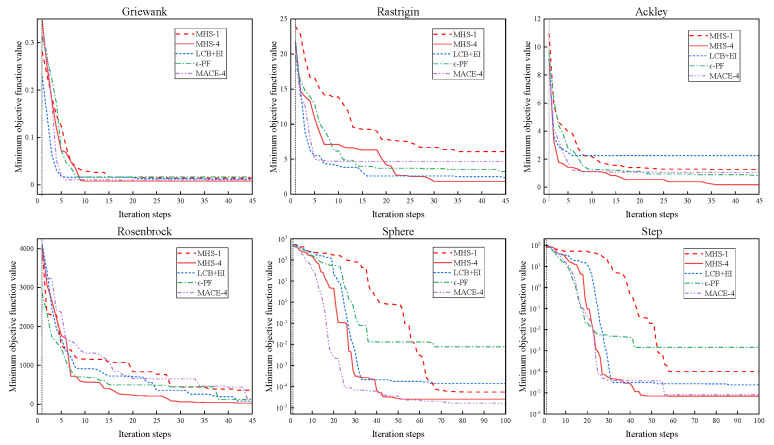
Average convergences of the MHS, EI + LCB, ϵ-PF, and MACE on benchmark functions.

In 10 independent runs, the standard deviation of optimizing benchmark functions by the test algorithm is shown in Figure 12. The average convergence of the test algorithm on the benchmark function is shown in Figure 13. Under parallel testing, the MHS performs well in the tests of Ackley, Griewank, Rastrigin, Sphere, and Step. Compared with LCB + EI and ϵ-PF, the MHS shows stronger accuracy and robustness. For example, in the tests of Ackley and Sphere, LCB + EI and ϵ-PF converge prematurely, while MHS-4 finds a better global solution. MHS features higher accuracy compared with MACE. In sequential mode, the MHS-4 shows a significant acceleration advantage compared with MHS-1. For example, as the target minimum value reaches 10^−3^ in the optimization of Sphere, the number of iteration steps of MHS-4 is 30 while that of MHS-1 is 65. In summary, the MHS is more competitive than other algorithms in parallel mode, both in terms of accuracy and speed.

#### 4.3.2. Effect of Time Range on Inversion Results

**Figure 14:**
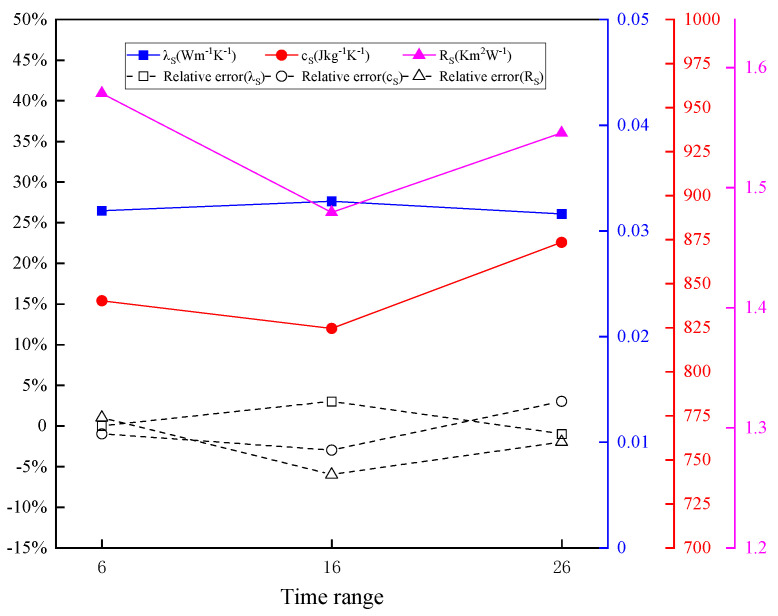
Results of thermophysical parameter inversion of GW in different time ranges.

**Figure 15:**
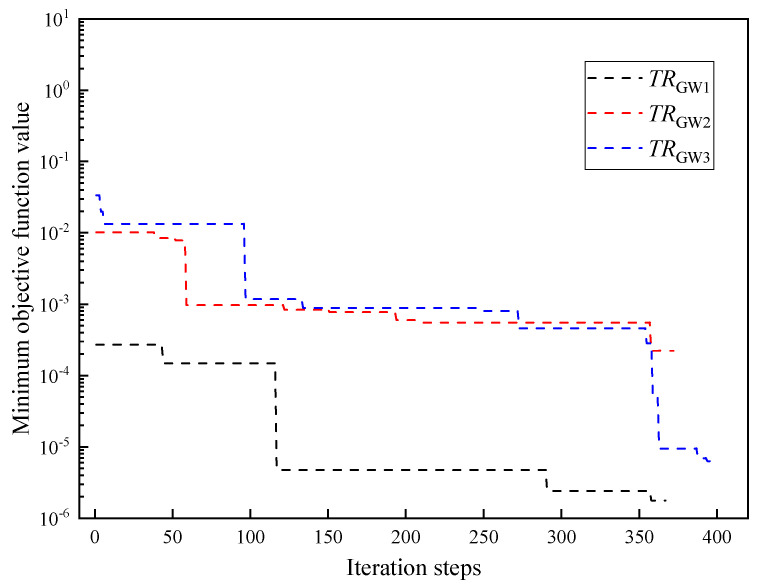
Iteration curves of parameter inversion in different time ranges.

The Latin hypercube method is adopted to conduct initial sampling in the decision space. The initial sample parameters are input into the TPS direct model for calculation. Three kinds of time range are selected for parameter inversion, where ${TR}_{\mathrm{GW}1}=6\;s$, ${TR}_{\mathrm{GW}2}=16\;s$, and ${TR}_{\mathrm{GW}3}=26\;s$. The inversion results of thermophysical parameters of GW in different time ranges are shown in Figure 14. It shows that the changes in time intervals have little influence on the inversion accuracy of the thermal conductivity, specific heat capacity, and thermal contact resistance. The iteration curves of parameter inversion in different time ranges are shown in Figure 15. The final minimum objective function values of the MHS for all time ranges are between 10^−3^ and 10^−6^.

#### 4.3.3. Effect of Initial Sample Number on Inversion Results

PSF is selected as the specimen to be tested. Three initial samples with different numbers are sampled in the decision space shown in Table 3 by the Latin hypercube method. The number initial sample is $M_{\mathrm{PSF}1}=70$, $M_{\mathrm{PSF}2}=140$, and $M_{\mathrm{PSF}3}=210$. Other settings remain unchanged.

**Figure 16:**
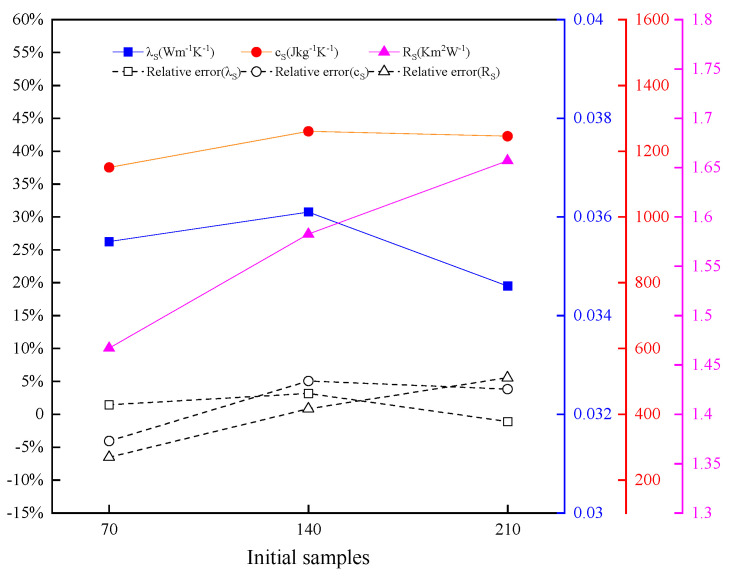
Results of thermophysical parameter parameters inversion of PSF under different initial sample numbers.

**Figure 17:**
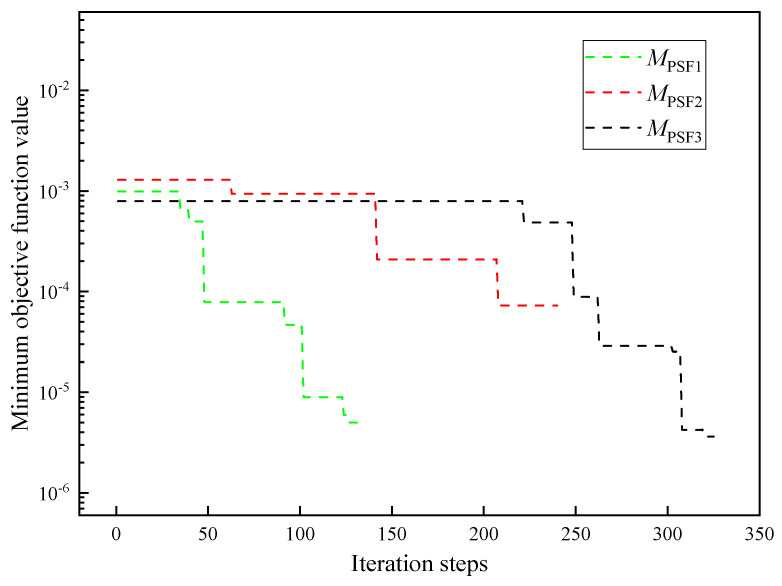
Iterative curve of parameter inversion of PSF under different initial sample numbers.

The results of the thermophysical parameters inversion of PSF under different initial sample numbers are shown in Figure 16. The relative errors between the inversion values of thermal conductivity, specific heat capacity, and thermal contact resistance under different initial sample numbers are small, whose absolute values remain within 3.61%, 5.05%, and 6.56%, respectively. The number of initial samples slightly influences the accuracy of the inversion. The iterative curve of parameter inversion of PSF under different initial sample numbers is shown in Figure 17. The minimum objective function values vary between 10^−4^ and 10^−6^.

#### 4.3.4. Effect of Parallel Points on Inversion Results

XPS is tested to investigate the effect of parallel points on parameter inversion results. The number of parallel points is $B_{\mathrm{XPS}1}=2$, $B_{\mathrm{XPS}2}=4$, and $B_{\mathrm{XPS}3}=6$. The other settings remain unchanged.

**Figure 18:**
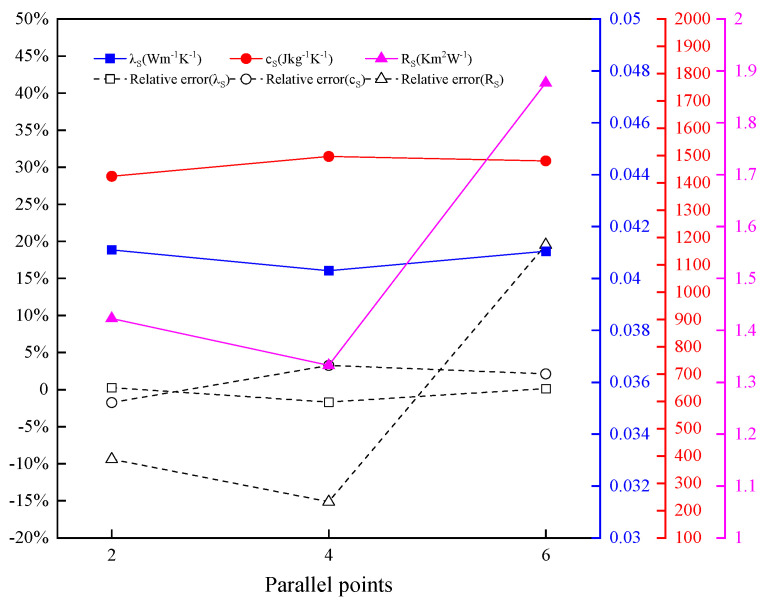
Inversion results of thermophysical parameters of XPS under different parallel points.

**Figure 19:**
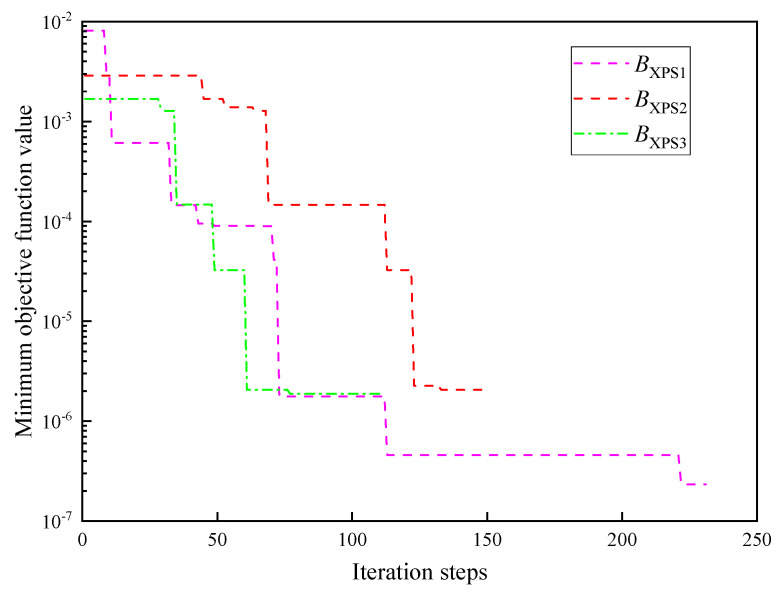
Iterative curve of parameter inversion of XPS under different parallel points.

The inversion results of the thermophysical parameters of XPS under different parallel points are shown in Figure 18. The inversion results of thermal conductivity and thermal contact resistance are still close to the real values. The errors indicate that the number of parallel points slightly influence accuracy. The iterative curve of parameter inversion of XPS under different parallel points is shown in Figure 19. The minimum objective function value remains between 10^−5^ and 10^−7^. When the number of parallel points is 2, 4, and 6, the minimum number of iterative convergence steps required for convergence is 231, 148, and 77, respectively. The time required to reach convergence gradually shrinks. With the increase in the number of parallel points, the speed of iteration is improved effectively, while accuracy is guaranteed. The high efficiency of the parallel strategy in the MHS is verified.

## 5. Conclusions

In this study, the mesh generation technology of the body-fitted coordinate system is used to convert the irregular structure into a regular grid, improving the calculation speed of the thermal conductivity model for the direct problem of the winding probe, and the parameter identification of the inverse heat transfer problem is carried out by the MHS. The main conclusions are drawn as follows:

(1) The selection of different angles has an influence on the calculation results, which is related to the orthogonality and density of the grid. After simulation analysis, it is determined that the partition angle at 30° is the best.
(2) The accuracy of the constructed direct problem algorithm and the reliability of the CFD results are verified. The absolute value of the relative error to the CFD calculation results is less than 0.03%, indicating the correctness of the direct problem algorithm. The BFG generation method is used to realize the structure of the model. The solution speed of the model application BFG is more than 36% and 91% higher than that of CFD and self-developed unstructured mesh programs, respectively.
(3) The MHS is compared with EI + LCB, ε-PF, and MACE on six standard functions. It shows a significant acceleration advantage in parallel mode. The accuracy and efficiency of the MHS are verified.
(4) The inversion results are slightly affected by time range, the number of initial samples, and parallel points. The optimization efficiency is improved by adding parallel points.
(5) Only low thermal conductivity insulation materials were considered in this study, and future research can be extended to medium and high thermal conductivity materials.

## Figures and Tables

**Table 1:** Geometric parameters and thermophysical properties of physical model.

		Heat Source	Coating	Insulation Layer	Thermal Resistance Layer	Sample
Material		Copper	Epoxy resin	Polyimide	/	GW/PSF/XPS
Geometric parameter		nu=81	$b_{\mathrm{cl}}=0.005$	$W_{\mathrm{il}}=0.5$	$H_{t}=0.038$	$W_{s}=50$
		$r_{w}=0.015$	$b_{\mathrm{cm}}=0.01$	$W_{\mathrm{ir}}=3$	/	$H_{s}=50$
		$r_{\mathrm{pr}}=3.755$	$H_{c}=0.02$	$H_{i}=0.075$	/	/
Thermophysical property	λ	398	0.168	0.168	0.0242	0.0318/0.035/0.041
	$c_{p}$	385	1100	1100	1006.43	850/1200/1450
	ρ	8960	1450	11,450	1.225	73/35/35

**Table 2:** Mathematical model.

Governing Equation [28]	Boundary Condition	Initial Condition		
$\begin{array}{r} \rho c_{p}\frac{\partial T}{\partial t}=\frac{1}{r}\frac{\partial }{\partial r}(\lambda r\frac{\partial T}{\partial r})+\frac{\partial }{\partial z}(\lambda \frac{\partial T}{\partial z})\\ +S(x,y) \end{array}$	Axis symmetry: $\frac{\partial T}{\partial r_{\mathrm{bound}1}}=0$ Symmetry: $\frac{\partial T}{\partial z_{\mathrm{bound}2}}=0$ Adiabatic [7]: $\frac{\partial T}{\partial z_{\mathrm{bound}3}}=0,\frac{\partial T}{\partial r_{\mathrm{bound}4}}=0$	Sample	$t_{p}$	$P_{h}$
		GW	30	0.004
		PSF	20	0.005
		XPS	20	0.005

**Table 3:** The initial parameters range.

Parameter	Lower Limit	UPPER Limit
λ	0.001	1
$c_{p}$	10	2500
$\delta_{t}$	0.001	0.1

**Table 4:** Time step for samples.

Material	Time Step
GW	0.02
PSF	0.01
XPS	0.01

**Table 5:** Time intervals of the different materials.

Material	t/θ Scope	t Scope
GW	[0, 1]	[0, 27.6]
PSF	[0, 1]	[0, 17]
XPS	[0, 1]	[0, 17.5]

**Table 6:** Dimensionality and search range of benchmark functions.

Test Function	Dimension d	Search Scope
Ackley	2	${\left[-18,18\right]}^{d}$
Griewank	3	${\left[-5,2\right]}^{d}$
Rastrigin	2	${\left[-10,10\right]}^{d}$
Rosenbrock	3	${\left[-10,10\right]}^{d}$
Sphere	8	${\left[-25,22\right]}^{d}$
Step	8	${\left[-10,10\right]}^{d}$

## Data Availability

Data are contained within the article.

## Nomenclature and Abbreviations

**Nomenclature**		$\alpha_{0},\gamma$	The metric coefficients in η,ξ directions
nu	Number of turns	$\beta_{x}$	The orthogonality of physical plane grid
$r_{w}$	Radius of the hot wire, mm	ω	Angle, °
$r_{\mathrm{pr}}$	Radius of the probe, mm	κ	Thermal diffusivity, $m^{2}\cdot s^{-1}$
$b_{\mathrm{cl}}$	Distance between the left side of the coating and the hot wire, mm	*θ*	Characteristic time, s
$b_{\mathrm{cm}}$	Minimum distance between two turns of copper wire, mm	τ	Dimensionless time
$W_{s}$	Width of sample, mm	σ	Temperature coefficient of resistance
$W_{\mathrm{il}}$	Width of insulation on left side of coating, mm	α, β	Strategy of adding parallel points
$W_{\mathrm{ir}}$	Width of insulation on right side of coating, mm	μ(x)	Average function
H	Height, mm	σ(x)	Variance function
δ	Thickness, mm	**Superscript**	
$c_{p}$	Specific heat capacity, $J\cdot {\mathrm{kg}}^{-1}\cdot K^{-1}$	0	Last time step
hc	Thermal contact resistance, $m^{2}\cdot K\cdot W^{-1}$	**Subscripts**	
T	Temperature, K	bound	Boundary
t	Time, s	t	Thermal resistance layer
$t_{p}$	Heating time, s	h	heat source
Δh	The heat flow distance	pr	Probe
$t_{\mathrm{tol}}$	total heating time, s	c	Coating
$P_{h}$	Heating power, W	i	Insulation layer
*S*	Heat source term in the physical plane, $W\cdot m^{-3}$	s	Sample
r,z	Horizontal and vertical coordinates of the cylindrical coordinate system in the physical plane	n,s,e,w	The upper, lower, right, and left edge centers of the solution grid, respectively
x,y	Horizontal and vertical coordinates of rectangular coordinate system in the physical plane	N,S,E,W	The center of the upper, lower, right, and left units of the solved grid, respectively
P,Q	Control functions	p	Center of the solution unit
J	Jacobi factor	**Abbreviations**	
R	Heat source term in the computational plane, $W\cdot m^{-3}$	BFG	Body-fitted grid
$\Delta \bar{T}$	Actual average temperature rise in probe heat source, K	TPS	Transient plane source
ΔT	Predicted average temperature rise in probe heat source, K	CFD	Computational fluid dynamics
l	An arbitrary parameter	GW	Glass wool
Δp	Change in parameter	PSF	Polystyrene foam
m	Time step	XPS	Extruded polystyrene
f	Objective function	MHS	Parallel Bayesian optimization
D	Variable dimension	q-EI	Multi-points expected Improvement
$R_{p}$	Probe resistance, Ω	EI	Expected improvement
R0	Probe initial resistance, Ω	GP	Gaussian process
**Greek symbols**		LCB	Lower confidence bound
λ	Thermal conductivity, $W\cdot m^{-1}\cdot K^{-1}$	MACE	Multi-objective acquisition ensemble
ρ	Density, $\mathrm{kg}\cdot m^{-3}$		
ξ,η	Horizontal and vertical coordinates in the computational plane		
